# Carbon fiber-reinforced pedicle screws reduce artifacts in magnetic resonance imaging of patients with lumbar spondylodesis

**DOI:** 10.1038/s41598-020-73386-5

**Published:** 2020-09-30

**Authors:** Christoph Fleege, Marcus Makowski, Michael Rauschmann, Katharina Luise Fraunhoffer, Peter Fennema, Mohammad Arabmotlagh, Marcus Rickert

**Affiliations:** 1 Orthopaedische Universitaetsklinik Friedrichsheim gGmbH, Marienburgstrasse 2, 60528 Frankfurt am Main, Germany; 2 Department of Radiology and Neuroradiology, Charité-University, Charitéplatz 1, 10117 Berlin, Germany; 3 Klinik für Wirbelsäulenorthopädie und Rekonstruktive Orthopädie, Sana Klinik Offenbach, Starkenburgring 66, 63150 Offenbach am Main, Deutschland; 4 AMR Advanced Medical Research GmbH, Hofenstrasse 89b, 8708 Männedorf, Switzerland

**Keywords:** Diseases, Medical research

## Abstract

The study investigated whether the use of carbon fiber-reinforced PEEK screw material (CF-PEEK) can reduce magnetic resonance imaging (MRI) artifact formation. Two consecutive groups of patients were treated for degenerative spinal disorders of the lumbar spine with dorsal transpedicular spinal fusion. The first group (n = 27) received titanium pedicle screws. The second group (n = 20) received CF-PEEK screws. All patients underwent an MRI assessment within the first four postoperative weeks. For each operated segment, the surface of the artifact-free vertebral body area was calculated as percentage of the total vertebral body. For each implanted segment, the assessability of the spinal canal, the neuroforamina, and the pedicle screws, as well as the surrounding bony and soft-tissue structures was graded from 1 to 5. A mean artifact-free vertebral body area of 48.3 ± 5.0% was found in the in the titanium group and of 67.1 ± 5.6% in the CF-PEEK group (*p* ≤ 0.01). Assessability of the lumbar spine was significantly improved for CF-PEEK screws (*p* ≤ 0.01) for all measurements. CF-PEEK pedicle screws exhibit smaller artifact areas on vertebral body surfaces and their surrounding tissues, which improves the radiographic assessability. Hence, CF-PEEK may provide a diagnostic benefit.

## Introduction

Pedicle screw fixation for spinal stabilization is now commonplace in spine surgery^1^. Currently, there are several implants available that are manufactured from various materials. The most popular orthopedic materials are metals such as titanium and its alloys^2^. After lumbar spondylodesis, it is vital that postoperative imaging can reliably be performed for diagnostic purposes. However, a drawback of metallic implants are the metal-induced artifacts on magnetic resonance imaging (MRI), resulting in signal loss, pile-up artifact and geometric distortion^3^. MRI is commonly used for the assessment of neural and soft-tissue structures (i.e., spinal canal, neuroforamina, and surrounding anatomical structures). Artifacts on MRI may lead to incorrect diagnosis, with potential serious consequences on patients’ health and outcomes^4,5^. Although methods such as Slice Encoding for Metal Artifact Correction (SEMAC)^6^ and Multi-Acquisition Variable Resonance Image Combination (MAVRIC)^7^ may improve image quality and decrease the extent of metal artifact, the utility of these methods as an appropriate diagnostic tool in symptomatic patients with metal implants still needs to be established^3^.

To overcome the aforementioned drawbacks of metallic implants, nonmetallic, high-strength, carbon fiber-reinforced polyetheretherketone (CF-PEEK) pedicle screws have been developed. CF-PEEK has a lower density than titanium (similar to bone), thus producing fewer artifacts in computed tomography (CT) and MRI^8–10^. Therefore, CF-PEEK would allow postoperative evaluation of fusion and adjacent segment disease, neural structures (in the event of developing neurological symptoms postoperatively), and spinal tumors.

A previously conducted study found that postoperative CT and MRI scans show reduced artifacts in patients who received CF-PEEK pedicle screws for the treatment of spinal tumors, when compared with standard titanium alloy implants^9^. To the best of the authors’ knowledge, there are no published studies comparing MRI artifacts of CF-PEEK and conventional titanium screws in the field of degenerative spine disease. Furthermore, there are no studies comparing the effect of CF-PEEK on the assessability of neural structures. The aim of the study was therefore to assess whether the use of CF-PEEK material can reduce MRI artifact formation and improve the assessability of important anatomical structures on imaging.

## Materials and methods

Two consecutive groups of patients were included in this retrospective cross-sectional study. All patients were operated on with dorsal transpedicular spinal fusion for the treatment of degenerative spinal disorders of the lumbar spine. Patients with traumatic (e.g., vertebral fractures) or inflammatory (e.g., spondylodiscitis, abscess, infections) conditions, tumor disease, or previous fusion surgery were not eligible for the study. The first group (n = 27) received titanium pedicle screws [Expedium Spine System (DePuy Synthes Spine Inc.)] and a PEEK cage (Medacta, Switzerland) (“titanium group”). The second group (n = 20) received CF-PEEK screws and a titanium-coated CF-PEEK cage (Icotec AG) (“CF-PEEK group”).

In the titanium group, patients were operated on between 2011 and 2017. An inclusion criterion was the availability of an MRI scan performed up to 4 weeks postoperatively. This was necessary for comparability, as in the CF-PEEK group standardized MRI examination took place up to 7 days postoperatively. In the CF-PEEK group, index surgeries took place from 2015 to 2017. All 47 study patients underwent dorsal transpedicular spondylodesis surgery for degenerative diseases of the lumbar spine. Baseline characteristics of the patients are presented in Table 1.

**Table 1:** Baseline characteristics of the study population.

Variable	Titanium group	CF-PEEK group	*p* value
Age (years)^a^	66.8 ± 16.9	54.3 ± 11.6	0.007
BMI (kg/m^2^)^a^	28.3 ± 4.2	26.3 ± 4.8	0.113
Female/male	11/16	11/9	0.333
**Localization**			–
L2–L3	4	–	
L3–L4	2	–	
L4–L5	13	20	
L5–S1	9	–	
L5–L6	1	–	
L6–S1	1	–	
Single-level procedure	24	20	0.251
Two-level procedure	3	0	
**Approach (# of patients)**			< 0.001
TLIF	14	20	
PLIF	13	–	

Presented as number of patients, except.

^a^Presented as mean ± standard deviation.

In the titanium pedicle screw group, transforaminal lumbar interbody fusion (TLIF) was performed in 14 patients (51.9%) and posterior lumbar interbody fusion (PLIF) in 13 patients (48.1%). In contrast, in the CF-PEEK group, TLIF was performed in all 20 patients (100%).

A TLIF technique at L4–L5 was performed monosegmentally in all patients in the CF-PEEK group, whereas in the titanium pedicle screw group, monosegmental fusion operations were performed in 4 patients in L2–L3 (14.8%), in 2 patients in L3–L4 (7.4%), in 11 patients in L4–L5 (40.7%), and in 7 patients in L5–S1 (25.9%). Three bisegmental fusions were found in 2 patients in the segment L4–S1 (7.4%) and in one patient (3.7%) with lumbarization of S1 in L5–L6–S1.

### Imaging protocol

MR imaging was performed with a Philips Achieva 1.5 T unit (Philips Healthcare) equipped with a standard SENSE spine coil. All patients underwent the following T2-w Sequences: T2w-TSE-Sequence sagittal TR = 3500 ms, TE = 100 ms, FoV 300 × 300 mm, voxel Size 2D 0.9 × 1.25 mm, matrix 332 × 237 mm, slice thickness 4 mm, Sequence approx. scantime 2:58 min. T2w-TSE-Sequence transverse TR = 2500 ms, TE = 100 ms, FoV 200 × 200 mm, voxel Size 2D 0.75 × 0.9 mm, matrix 268 × 214 mm, slice thickness 3 mm, Sequence approx. scantime 2:55 min. Aforementioned T2-w sequences were chosen because they can be applied to the sagittal and tranversal section, and in our opinion this weighting is usually the best way to assess, intervertebral discs, paravertebral soft tissue, neural structures and the spinal canal.

### Outcomes assessment

All MRIs were performed within the first four postoperative weeks. Using current MRI reader software (Freehand 2D ROI measuring tool, Visage 7, Visage Imaging GmbH), one radiologist and a research fellow measured the artifact area on the scan of each treated level with titanium pedicle screws. The measurements started at the slice with the first artifacting reaction and ended with the last slice exhibiting an artifact reaction.

**Figure 1:**
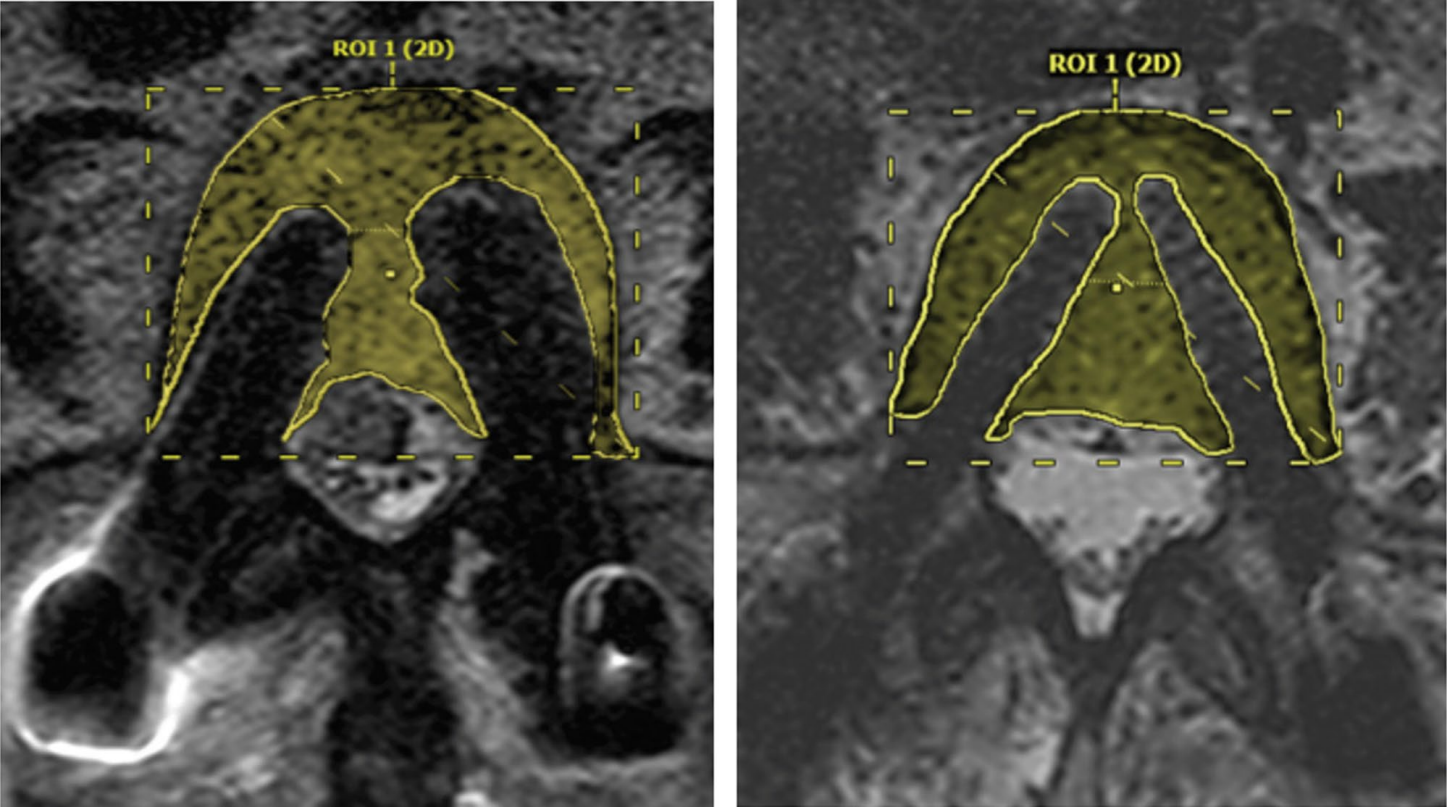
MRI transverse sections for comparison between the titanium group (left) and the PEEK group (right) in the calculation of the artifact-free area.

After image adjustment with selection and adaptation of the MRI section that was to be assessed, the vertebral body containing the pedicle screw implant was graphically reconstructed using the MRI reader software. The resulting area of the total vertebral body surface was calculated by the software. In a second computer-aided image processing, the vertebral body surface without both implants and the generated artifacts as the artifact-free vertebral body area were created (Fig. 1). Thereafter, a percentage calculation of the extent of the remaining vertebral body area excluding artifact phenomenon was made.

Next, another MRI evaluation of assessability of relevant anatomical structures in the surgical field was performed in the reader software. Assessability was graded from 1 to 5, where grade 1 allowed an “excellent assessability” without artifacts and grade 5 represented “no assessment possible” because of appearing artifacts (Table 2). For each measurement of assessability, three levels were taken, with Level 1 being the level of the cranial instrumented vertebra, Level 2 representing the position of the cage, and Level 3 defining the level of the caudal instrumented vertebra. In cases of bisegmental interventions, each investigation was extended with a further caudal screw segment.

**Table 2:** Classification of assessability.

Grade	Classification	Description
Grade 1	Very good	Synonymous with “excellent assessment” of anatomically relevant structures without any artifacting effect on the considered structure. Possible pathologies were clearly visible
Grade 2	Good	Due to slight irregularities in the image, sufficient assessment was feasible
Grade 3	Fair	Scattering effects of artifacts still allowed moderate assessment of anatomical structures, but image quality and certainty of assessment were reduced
Grade 4	Poor	Greatly reduced overall assessment and parts of anatomical structure were no longer clearly definable due to artifacts
Grade 5	Very poor	“Absolutely no judgment possible” in terms of anatomical consideration. The pictured area was no longer distinguishable from surrounding structures

The spinal canal, the neuroforamina, and the pedicle screws as well as the surrounding bony and soft-tissue structures were included in the assessability of the implanted segment. Assessment of the spinal canal was performed in each patient in the sagittal and transverse planes in the operated segments. The neuroforamina were evaluated in the operated segments in the sagittal plane. The assessment of the position of the pedicle screws was carried out in each patient side-by-side for each operated level. The surrounding soft tissue, including the residual tissue of the intervertebral disc with possible expansion into the spinal canal (herniation), the subcutaneous fat and the adjacent autochthonous back musculature with possible edema, seroma or hematoma formation, were analyzed for assessability. There was also a corresponding rating according to the above criteria. The assessability of the surrounding bony structures took place in the area of the vertebral body including the endplates with possible bone marrow oedema or osteophyte formation as well as the intervertebral joints with potential hypertrophy and constriction of neuronal structures.

For each of the structures, assessability was further analyzed by evaluating the statistical association with the size of the artifact-free area of the vertebral bodies.

Ethics committee approval was obtained prior to study commencement (Ethics Committee of the University Hospital Frankfurt, approval number 145/14) and all subjects provided informed consent.

### Statistical analysis

For continuous data, the Kolmogorov–Smirnov test was used to assess the normal distribution assumptions. Categorical variables are presented as frequencies and percentages. Continuous data are presented as mean and standard deviation (SD). Between-group comparisons were performed using the chi-square test or Fisher exact test for categorical variables, the Mann–Whitney test for ordinal variables, and the Student’s t-test or the Mann–Whitney test for continuous variables. Spearman rank correlation was used to evaluate the correlation between the artifact-free vertebral body surface and assessability of the spinal canal.

Statistical analyses were performed using SPSS Version 24 for Microsoft Windows (IBM Corp.). Two-sided tests were used throughout and *p* values ≤ 0.05 indicated statistical significance.

## Results

For the analysis of artifact-free vertebral body surfaces, one caudal vertebral body (1.8%) in the titanium pedicle screw group and one caudal vertebral body in the CF-PEEK group were excluded from the analysis due to insufficient quality of the MRI images. In the titanium group, the absolute area values of the vertebral bodies were 19.4 ± 5.89 cm^2^ and the absolute values of the artifact-free areas were 9.4 ± 3.66 cm^2^. As a percentage, the mean value of the artifact-free vertebral body area in the titanium pedicle screw group was 48.3 ± 5.0%. In the CF-PEEK group, the absolute value of the vertebral bodies was 16.1 ± 3.6 cm^2^ and the mean value of the artifact-free vertebral bodies was 10.9 ± 2.9 cm^2^. The percentage calculation showed a mean artifact-free vertebral body area of 67.1 ± 5.6% in this group. The difference in artifact-free vertebral body surfaces between the two groups was statistically significant (*p* ≤ 0.01).

**Figure 2:**
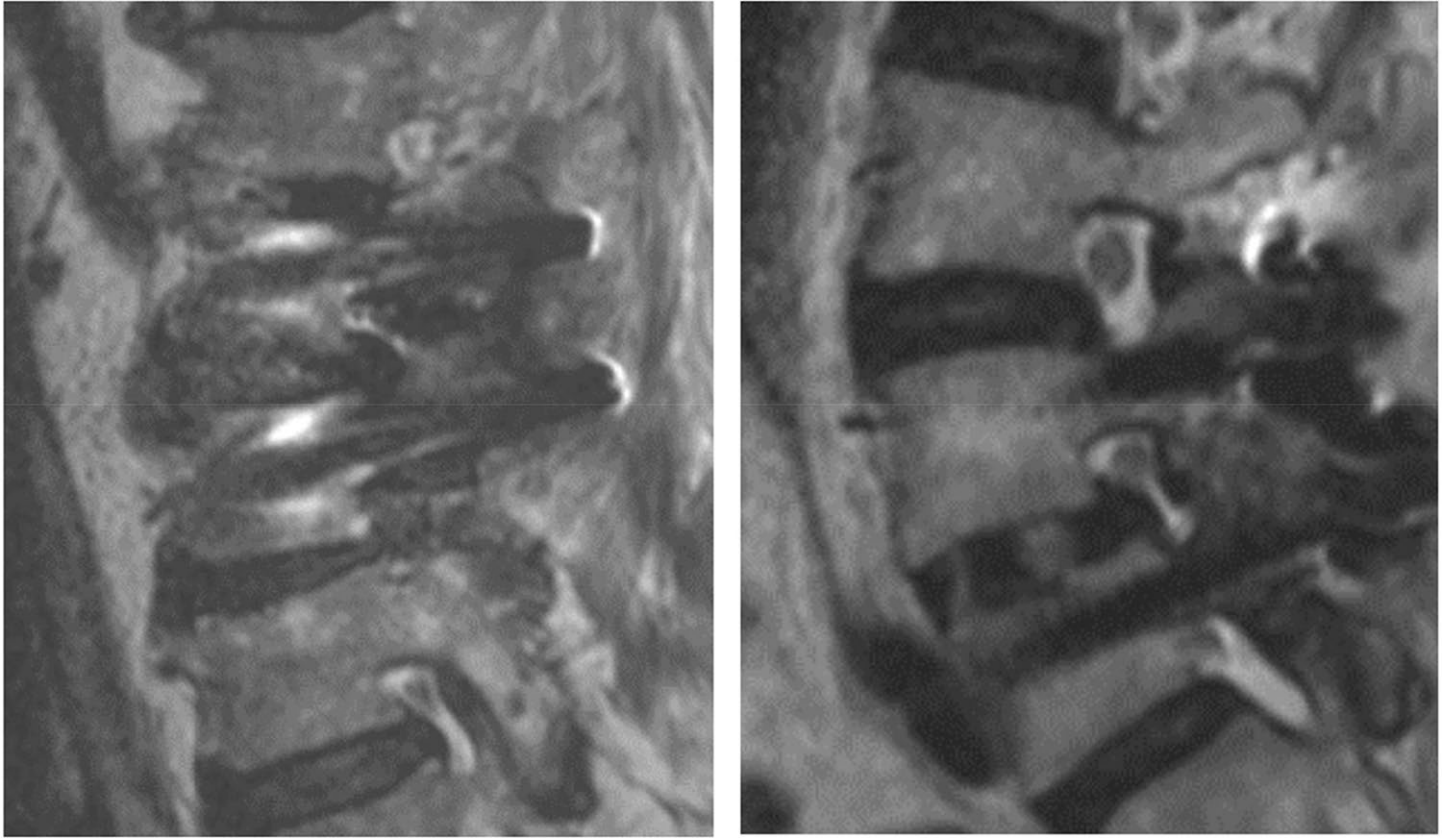
Comparison of the assessability of the neuroforamina at Level 2 (cage position). MRI images show a paramedian sagittal section for evaluating the neuroforamina. In the titanium group (left), the artifacts along the longitudinal axis of the screw were pronounced and the assessability of neuroforamina at the level of the cage was severely impaired. By comparison, there were no artifacts in the neuroforamina in the PEEK group (right).

In the titanium group, a mean degree of assessability of the spinal canal of 2.8 ± 0.7 was determined, and in the CF-PEEK group, the average grade of assessability was 1.4 ± 0.5 in the range of grade 1 (“very good”) and grade 2 (“good”) (*p* ≤ 0.01). The mean assessability of all neuroforamina was assessed to be 2.8 ± 0.6 in the titanium group and 1.3 ± 0.3 in the CF-PEEK group (*p* ≤ 0.01). In Level 1 and Level 3, the assessability was 2.3 ± 0.9 and 1.2 ± 0.4 for the titanium group and the CF-PEEK group, respectively (*p* ≤ 0.01). When comparing the assessability of the neuroforamina in Level 2 (cage position), it was 3.8 ± 0.9 in the titanium group and 1.6 ± 0.7 in the CF-PEEK group (*p* ≤ 0.01). Compared with Levels 1 and 3, the assessability of the neuroforamina was significantly lower. Furthermore, assessability of Level 2 was significantly lower in the titanium pedicle screw group (*p* ≤ 0.01) (see Fig. 2).

The calculation of the Spearman rank correlation showed a negative correlation between the assessability of the neuroforamina and the absolute values of the artifact-free areas, with a correlation coefficient r_s_ = −0.750 (*p* ≤ 0.01).

Assessability of the pedicle screws was 3.0 ± 0.6 and 1.3 ± 0.3 in the titanium group and CF-PEEK group, respectively (*p* ≤ 0.01). For the association between the absolute values of the artifact-free areas and assessability of the titanium pedicle screws, a negative correlation was found: r_s_ = −0.770 (*p* ≤ 0.01).

In assessing soft tissues (musculature and ligamentous apparatus), the recognizability of possible structural changes and pathological abnormalities found a mean assessability of soft-tissue structures in the surgical field of 3.3 ± 0.6 in the titanium group and of 1.6 ± 0.6 in the CF-PEEK group (*p* ≤ 0.01).

The assessment of the assessability of the bony segment area found a mean value of 4.3 ± 0.5 in the titanium group and of 2.2 ± 0.6 in the CF-PEEK group (*p* ≤ 0.01).

## Discussion

The main finding of the study was that the use of CF-PEEK pedicle screws results in a substantial reduction of MR artifact areas on vertebral surfaces, which significantly improved the postoperative assessability of anatomical structures through MRI after lumbar spondylodesis, and thus provides a substantial diagnostic benefit.

CF-PEEK pedicle screws have only recently become available, and PEEK and reinforced (neat) PEEK composites as a material for screw/rod constructs have been suggested to have the potential to overcome major drawbacks of standard titanium^10^.

A previously conducted study assessed the difference between CF-PEEK and titanium pedicle screws in patients treated for spinal tumors^9^. This study found a 52% reduction of image artifacts surrounding pedicle screws on CT in patients with CF-PEEK pedicle. However, these conclusions are not necessarily generalizable to the area of degenerative spine disease as the study was limited to the assessment of intraosseous artifacts. Whereas such a focus is meaningful for the planning of radiotherapy in oncologic patients, a focus on intraosseous artifacts does not suffice in patients with degenerative disease of the spine. For example, postoperative neurological deficits are common and often the cause of uncertainty in postoperative MRI with titanium implants. In that case, a radiologic assessment may not be able to differentiate between hematoma and residual stenosis. With preservation of relevant imaging information due to hardware composed material with lower magnetizability, a better assessability of the spinal canal and the neuroforamen can be achieved.

This retrospective study was performed to compare the artifacts and the anatomical relevance of structures in postoperative MRI images after spinal fusion. Titanium alloy implants were compared to CF-PEEK.

With consideration of the inherent limitations of this report, retrospective studies are susceptible to several forms of bias, including selection bias, and causal inferences must be interpreted cautiously. For the present study, the critical assessment of retrospective studies was counteracted in the present analysis by consecutive enrolment of patients and by ensuring a prospective documentation of the database with regard to the clinical and radiological findings. This documentation of data was standardized during the observation period.

However, selection bias is likely to be present, which was shown in the between-group differences in the operated segment. The CF-PEEK pedicle screws were only recently introduced in our clinic, and mainly younger patients were selected in order to have as good a bone quality as possible with a material for which long-term results are lacking.

Whereas in the CF-PEEK group only L4–L5 was instrumented, several segments in the titanium pedicle screw group were treated. This difference in operated segment coincided with differences in surgical approach (100% TLIF in the CF-PEEK group and 51.9% TLIF in the titanium pedicle screw group). A total of 10 patients in the titanium group underwent surgery with involvement of the sacrum (7 patients at L5–S1, 2 at L4–S1, and 1 at L6–S1 in the form of a lumbarization). The transverse section through the sacrum shows a different morphology compared to the other lumbar vertebrae. It should be noted that the lumbar vertebrae L1–L5 caudally increase in size. The calculation of the mean artifact-free vertebral body surfaces, which was relative to the total surface of the vertebral body, is likely a source of bias. Hypothetically, an even larger difference between the two groups would come to bear with a standardized height localization of the vertebral body fusion performed and with a more homogeneous group composition with regard to shape and size of the vertebral bodies. In the next investigation, such an implementation must be considered.

Comparative quantitative determinations of the artifact level between metal and CF-PEEK implants are scant. In an in vitro study from 2012, a comparison of artifact formation in MRI images was followed by a study of plate osteosynthesis with a material from CF-PEEK/tantalum as well as from titanium on cadaver bones^11^. In the area of the screws introduced at the humeral head, the length of the artifacts perpendicular to the screws was determined. Artifact lengths measured in titanium implants were 9.7 ± 3.1 mm, 3.6 ± 0.5 mm for CF-PEEK/tantalum beads, and 4.0 ± 0.8 mm for PEEK/tantalum fibers. The authors concluded that CF-PEEK implants are an alternative to titanium plates due to the significantly reduced artifact imaging within the field of application in traumatology and tumor orthopedics.

To conclude, the study results suggest that CF-PEEK pedicle screws exhibit fewer artifact areas on vertebral body surfaces and on the surrounding tissues, which significantly improves the radiographic assessability of anatomical structures in the surgical site after lumbar spondylodesis. Therefore, CF-PEEK can provide a substantial diagnostic benefit in detecting potential complications.
